# Assessment of testicular volume in neonates in the tropical province of China

**DOI:** 10.1186/s12887-023-04274-w

**Published:** 2023-09-02

**Authors:** Shaohua Hu, Zhenli Zhao, Zhisheng Wan, Weizhen Bu, Songqiang Chen, Shuai Yang, Xiaowen Chen, Yiqun Lu

**Affiliations:** 1 Department of Urology, Hainan Women and Children’s Medical Center, Changbin Road, Xiuying District, Haikou, 570206 China; 2 Department of Ultrasound, Hainan Women and Children’s Medical Center, Changbin Road, Xiuying District, Haikou, 570206 China; 3 Department of Pediatric, Hainan Women and Children’s Medical Center, Changbin Road, Xiuying District, Haikou, 570206 China; 4 Department of Urology, Children’s Hospital of Fudan University, No 399, Wanyuan Road, Minhang District, Shanghai, 201102 China

**Keywords:** Neonates, Premature, Low birth weight, Testicular volume, Cryptorchidism

## Abstract

**Background:**

Testicular volume in neonates is a potential indicator of testicular development during the fetal period, particularly the masculinization programming window. Reliable measurements of testicular volume provide an opportunity for early detection of testicular abnormalities. This study aimed to assess the testicular volume in neonates and evaluate its relationship with gestational week and birth weight in Hainan Province, China.

**Methods:**

Data on 458 neonates who underwent ultrasonography examinations at our institution from 2018 to 2022 were collected. The neonates were categorized by gestational week, birth weight, and presence of cryptorchidism. We evaluated the testicular volume among different groups and its relationship to gestational week and birth weight.

**Results:**

There was no significant difference between the right and left testicular volume in neonates without cryptorchidism. However, a significant difference was observed between normal birth weight and low birth weight neonates in terms of testicular volume. Similarly, there was a significant difference between premature and full-term neonates in testicular volume. Bilateral testicular volume showed positive and significant correlations with gestational week and birth weight. Additionally, a significant difference was noted in testicular volume between the affected side in neonates with cryptorchidism and the same side in normal birth weight full-term neonates.

**Conclusions:**

We established the normal range of testicular volume for neonates in Hainan Province and demonstrated that testicular volume is positively correlated with both birth weight and gestational week. Cryptorchidism also affects testicular volume during the neonatal period, likely due to reduced androgenic exposure in utero, particularly during the masculinization programming window. The findings of this study have significant implications for assessing testis development during fetal development.

## Background

Evaluating the reproductive system is a crucial component of neonatal physical examination, enabling early detection and treatment of potential abnormalities. Testicular volume is associated with various reproductive endocrine parameters, such as serum follicle-stimulating hormone (FSH), serum statin B, statin B/FSH ratio, and serum testosterone, which are directly or indirectly related to spermatogenesis. As spermatogenic tubules account for 80% ~ 90% of the testicular mass, measuring testicular volume reflects spermatogenesis to a considerable extent. Therefore, assessing testicular volume is vital for evaluating testis function [1, 2].

The fetal testis secretes testosterone, which is necessary for male newborn virility and continued penile growth. The assessment of testicular volume at birth is essential for the early detection of sexual development disorders, such as gonadal dysplasia and/or androgen synthesis disorders. This makes testicular volume in neonates clinically predictive, reinforcing the need to establish normal testicular volume at birth [3]. However, there are no reports on measuring testicular volume in normal and cryptorchidism neonates in the tropical province of China. Although there are some studies abroad, it remains unclear whether there are differences due to the influence of race, geographical environment, social economy, and other factors. Therefore, this study measured testicular volume using ultrasonography in neonates in Hainan Province, China, and evaluated its relationship with gestational week and birth weight, to provide a reference for early clinical diagnosis of testis-related diseases in neonates.

## Materials and methods

### Patient summary and ethical approval

Data on 458 neonates who underwent ultrasonography examinations at our institution from 2018 to 2022 were collected. This is the data of all neonates who underwent ultrasonography that met the inclusion criteria, therefore, this research did not involve sampling and no sample size calculations were performed. Inclusion criteria: neonates within 28 days of birth, without penile and testicular diseases such as hypospadias, epispadias, testicular tumors, abnormal sexual development, etc.

The neonates were categorized by gestational week, birth weight, and presence of cryptorchidism: normal birth weight full-term neonates (n = 300), low birth weight full-term neonates (n = 13), normal birth weight premature neonates (n = 24), low birth weight premature neonates (n = 56), left cryptorchidism neonates (n = 19), right cryptorchidism neonates (n = 11), bilateral cryptorchidism neonates (n = 35). The study was approved and the informed consent was waived by the Ethics Committee of Hainan Women and Children’s Medical Center., Haikou, China.

## Methods

According to the conditions at birth into different categories, ultrasonography measurement by experienced ultrasound specialists. Bilateral testes were measured the length, width, and thickness(cm), according to the formula of testicular volume: testicular volume = 0.7*length*width*thickness, computing the testicular volume of the neonates.

### Statistical analysis

SPSS 26.0 software was used for the statistical analysis of relevant data. The measurement data were expressed as mean ± standard deviation ($\overline{x}$±s). Testicular volume was normally distributed, and the t test was used. A nonparametric test was used when the normal distribution was not satisfied. Spearman linear correlation was used to analyze the correlation between testicular volume and birth weight and gestational week. P < 0.05 was considered statistically significant.

## Results

### Comparison of testicular volume in the left and right testis in each group

There was no significant difference in the volume of the left and right testis in normal birth weight full-term neonates, low birth weight full-term neonates, normal birth weight premature neonates, and low birth weight premature neonates (the t values were 1.209, 1.256, 0.201, 0.452, P > 0.05), as shown in Table 1.

**Table 1:** Comparison of testicular volume in the left and right testis in each group (cm^3^, $\bar x$±s)

Group	n	Age	Birth weight	Gestational week	Left testis	Right testis	t	P
Normal birth weight full-term neonates	300	2(1,28)	3399 ± 420	39.3 ± 0.9	0.38 ± 0.14	0.383 ± 0.139	1.2	0.2
Low birth weight full-term neonates	13	6(1,22)	2169 ± 225	37.8 ± 2.7	0.28 ± 0.09	0.24 ± 0.06	1.3	0.2
Normal birth weight premature neonates	24	6(1,28)	2878 ± 220	35.7 ± 1.6	0.27 ± 0.1	0.28 ± 0.1	0.2	0.8
Low birth weight premature neonates	56	7(1,28)	1953 ± 335	34 ± 2.2	0.23 ± 0.1	0.22 ± 0.1	0.5	0.7

### Comparison of testicular volume between normal birth weight and low birth weight neonates

There was a significant difference between normal birth weight full-term neonates and low birth weight full-term neonates in bilateral testicular volume (the t values were 2.582, 7.517, P < 0.05), and there was also a significant difference between normal birth weight full-term neonates and normal birth weight premature neonates in bilateral testicular volume (the t values were 3.666, 3.657, P < 0.05), as shown in Table 2.

**Table 2:** Comparison of testicular volume in low birth weight and premature with normal birth weight full-term neonates respectively (cm^3^, $\bar x$±s)

Group	n	Left testis	t	P	Right testis	t	P
Low birth weight full-term neonates	13	0.28 ± 0.09	2.6	0.01	0.24 ± 0.06	7.5	< 0.001
Normal birth weight full-term neonates	300	0.38 ± 0.14			0.38 ± 0.14		
Normal birth weight premature neonates	24	0.27 ± 0.1	3.7	< 0.001	0.28 ± 0.1	3.7	< 0.001

### Evaluation of testicular volume in relation to gestational week and birth weight

Bilateral testicular volume in neonates was positively correlated with birth weight and gestational week (birth weight: the r values were 0.5, 0.49, P < 0.05; gestational week: the r values were 0.36, 0.37, P < 0.01), as shown in Table 3.

**Table 3:** Testicular volume in relation to gestational week and birth weight

			Birth weight	Gestational week
Testicular volume	Left	r	0.5	0.36
		P	< 0.01	< 0.01
	Right	r	0.49	0.37
		P	< 0.01	< 0.01

### Comparison of testicular volume between the affected side of neonates with cryptorchidism and the same side of normal neonates

There was a significant difference between the affected side of neonates with cryptorchidism and the same side of normal neonates (left cryptorchidism, the Z value was 4.571, P < 0.05; right cryptorchidism, the Z value was 2.467, P < 0.05; bilateral cryptorchidism: the Z values of the left and right side were 6.254, 6.367, P < 0.05), as shown in Tables 4 and 5.

**Table 4:** Comparison between normal ipsilateral testes and cryptorchidism of right and left testes(cm^3^)

Group	n	Left testis	Z	P
Left cryptorchidism	19	0.22(0.2–0.3)	4.6	< 0.001
Normal neonates	300	0.35(0.29–0.45)		
Right cryptorchidism	11	0.18(0.17–0.41)	2.5	0.014

**Table 5:** Comparison of testicular volume between bilateral cryptorchidism and normal neonates (cm^3^)

Group	n	Gestational week	Left testis	Z	P	Right testis	Z	P
Normal neonates	300	39.3 ± 0.9	0.35(0.29–0.45)	6.3	< 0.001	0.36(0.29–0.45)	6.4	< 0.001
Bilateral cryptorchidism	35	35.8 ± 3.1	0.21(0.16–0.28)			0.23(0.17–0.3)		

## Discussion

Cryptorchidism is one of the most common congenital developmental malformations of the urogenital system in children, with an incidence of 1–4% in full-term neonates and 30% in premature neonates, moreover, the incidence of cryptorchidism is as high as 60–70% for very low birth weight neonates (birth weight less than 1500 g) [4, 5]. Preterm birth and low birth weight are recognized as two important risk factors for congenital cryptorchidism [6]. Physical examination, particularly of genitalia, is crucial for all neonates. Deviations in the size of external genitalia could be the initial indication of underlying endocrine or genetic disorders, making it a valuable diagnostic tool [7]. Testicular volume is an important indicator of external genitalia androgenization and reflects the activity and normality of the hypothalamic-pituitary-testicular axis. It is closely linked to abnormal genital development and androgen deficiency in male neonates. Thus, determining the correct testicular volume at birth is crucial in assessing the degree of masculinity of the external genitalia [8–10]. Unfortunately, there are few studies on neonatal testicular volume in China, and none have been conducted in Hainan Province.

The Prader orchidometer is traditionally used for measuring testicular volume, but it has two main problems. First, when measuring testicular volume, it is important to note that the smallest bead in the Prader orchidometer is 1 cm3, while ultrasonography measurement does not exceed 0.44 cm3 in the first few years of life. Second, the orchidometer may overestimate testicular volume in cases where the epididymis is larger than the testis, as it measures both the epididymis and scrotal skin. This can reduce sensitivity and make the measurement unrepeatable. [8, 11]. Ultrasonography is a more effective method to measure testicular volume in small testis as it can detect even minor biological changes associated with physiological changes during the neonatal period and the first year of life. In addition, it is also capable of detecting relevant abnormalities in the testis and other structures within the scrotum and has the advantages of being objective, accurate, repeatable, and does not involve ionizing radiation. Therefore, it can be used to further evaluate the testis of neonates [12]. Based on the simplicity and accuracy of ultrasonography, we used it to measure the testicular volume of neonates in Hainan Province.

In our study, we first measured the testicular volume of full-term normal-weight neonates. The results showed no significant difference in the volume of the left and right testis. The volume of the left testis was 0.377 ± 0.138 cm^3^ and that of the right testis was 0.383 ± 0.139 cm^3^. Ogundoyin, Brandt, Chin, and Ting respectively measured the testicular volume of male neonates in the typical African population (Nigeria), white and black population, Taiwan district, Malaysia, and Chinese by Prader orchidometer, the mean testicular volume was 1.14 ± 0.38 cm^3^, 1.1 ± 0.6 cm^3^ (white and black population), 1.3 ± 0.3 cm^3^, 2.5 ± 0.6 cm^3^ (Malaysia) and 2.4 ± 0.5 cm^3^ (Chinese), respectively [8, 13–15]. Semiz measured the testicular volume of Turkish neonates within 48 h after birth by Prader orchidometer, the volume of the left testis was 1.64 ± 0.68 cm^3^ and that of the right was 1.73 ± 0.45 cm^3^ [16]. Hagag measured the testicular volume of neonates at 1–4 weeks in Egypt by Prader orchidometer, the right testicular volume was 1.81 ± 0.44 cm^3^ and that of the left was 1.67 ± 0.47 cm^3^ [1]. The data measured by the above scholars are significantly higher than the testicular volume of neonates measured by us. However, Kaplan and Atalabi measured the testicular volume of neonates by ultrasonography, the result was 0.26 cm^3^ and 0.28 ± 0.09 cm^3^ respectively, which were lower than that measured by us [12, 17]. Logsdon dissected 32 fetuses from 10 to 22 gestational weeks who died of hypoxia and found that testes were all located in the abdominal cavity, the volume of the left testis was 17.25 mm^3^ and that of the right was 19.84 mm^3^. Correlation analysis showed that bilateral testicular volume increased significantly with gestational week [18]. Pires measured the testis from 35 normal fetuses who died between 11 and 22 gestational weeks, the volume of the left testis was 18.2 ± 13.91 mm3 and that of the right was 21.16 ± 17.54 mm3 [19]. We consider that the reasons for the above differences are firstly due to the error of the Prader orchidometer, which leads to the results measured by the Prader Orchidometer being larger than that by ultrasonography. Moreover, although the measurement results of the dead fetus by some scholars are smaller than that by ultrasonography, but significantly different from Prader orchidometer results, this proved that ultrasonography measurement of neonatal testicular volume is more objective and accurate. Secondly, the results are different due to factors such as geographical location, race, and social economy. Therefore, we need to establish a reference value for the normal testicular volume of male neonates in this area.

Next, we categorized the neonates according to birth weight, the results showed that there was a significant difference between normal birth weight and low birth weight neonates in bilateral testicular volume, normal birth weight neonates had larger testicular volume. The neonates were also categorized according to gestational week, the results showed that there was a significant difference between premature neonates and full-term neonates in bilateral testicular volume, and full-term neonates had larger testicular volume. Spearman correlation analysis of testicular volume with birth weight and gestational week indicated that testicular volume was positively correlated with birth weight and gestational week. This result had also been confirmed in other related studies [3, 20–22].

The production of gonadotrophin and testosterone decreases shortly after birth, then they begin to rise again to adolescent levels from about 1 week of age, reaching a peak at 1–3 months of age, dropping to pre-adolescent levels by 6 months of age at the last, so that spontaneous testicular decline can continue in the first few months of life [23]. However, there is no research on whether the testicular volume has been affected in neonatal cryptorchidism. In this experiment, we also measured the testicular volume of cryptorchidism during the neonatal period, the result showed that the testicular volume of the affected side of cryptorchidism was smaller than that of normal neonates on the same side, which had a significant difference, suggesting that the testicular volume of cryptorchidism had been affected in the neonatal period after birth, this may be related to reduced androgenic exposure in utero [24, 25].

Admittedly, there are some limitations to this study. First, the development of the penis is affected by testosterone, since the testicular volume of cryptorchidism had been affected in the neonatal period, whether the penis size of neonates with cryptorchidism is different from that of normal neonates has not been studied. Second, after 6 months, the affected testis in some children with cryptorchidism during the neonatal period will descend into the scrotum again, whether the testicular volume of the affected testis can catch up to the normal level after mini puberty needs further study.

## Conclusions

We established the normal range of testicular volume for neonates in Hainan Province and demonstrated that testicular volume is positively correlated with both birth weight and gestational week. Cryptorchidism also affects testicular volume during the neonatal period, likely due to reduced androgenic exposure in utero, particularly during the masculinization programming window. The findings of this study have significant implications for assessing testis development during fetal development.

## Data Availability

The datasets used and analyzed during the current study are available from the corresponding author on reasonable request.
